# Molecular Signature of Tumors with Monoallelic 13q14 Deletion: a Case Series of Spindle Cell Lipoma and Genetically-Related Tumors Demonstrating a Link Between FOXO1 Status and p38 MAPK Pathway

**DOI:** 10.1007/s12253-017-0303-6

**Published:** 2017-09-08

**Authors:** Karina Uehara, Fukino Ikehara, Ryo Shibuya, Iwao Nakazato, Mariko Oshiro, Masaya Kiyuna, Yasuka Tanabe, Zensei Toyoda, Kiyoto Kurima, Shinichiro Kina, Masanori Hisaoka, Takao Kinjo

**Affiliations:** 1 Division of Morphological Pathology, Department of Basic Laboratory Sciences, School of Health Sciences, University of the Ryukyus, 207 Uehara, Nishihara, Okinawa, 903-0215 Japan; 2 Department of Pathology and Oncology, School of Medicine, University of Occupational and Environmental Health, Fukuoka, Japan; 3 Department of Pathology, Okinawa Prefectural Nanbu Medical Center and Children’s Medical Center, Okinawa, Japan; 4 Health Information Management Major, Management and Information Science Division, Faculty of International Studies, Meio University, Okinawa, Japan; 5 Department of Pathology, Tomishiro Chuo Hospital, Okinawa, Japan; 6 Department of Regenerative Medicine, Graduate School of Medicine, University of the Ryukyus, Okinawa, Japan; 7 Department of Oral and Maxillofacial Functional Rehabilitation, Graduate School of Medicine, University of the Ryukyus, Okinawa, Japan

**Keywords:** Monoallelic 13q14 deletion, RB 1, FOXO 1, Reactive oxygen species, p38 MAPK

## Abstract

Spindle cell/pleomorphic lipomas (SCLs), cellular angiofibromas (CAFs) and mammary-type myofibroblastomas (MFBs) are rare benign mesenchymal tumors with monoallelic 13q14 deletion. They are predicted to have a common pathogenic mechanism due to shared similar histological and immunohistochemical features; however, pathological consequences of monoallelic 13q14 deletion remain unknown. We previously reported a CAF case with monoallelic 13q14 deletion in which the tumor expressed decreased levels of FOXO1 and RB1, both of which were encoded in 13q14, and increased reactive oxygen species (ROS) levels. We further demonstrated the activation of p38 mitogen-activated protein kinase (p38 MAPK) pathway induced by oxidative stress. We hypothesized that SCLs, CAFs and MFBs would share common molecular signatures involving FOXO1, ROS and p38 MAPK and that their expression patterns were different from those tumors without monoallelic 13q14 deletion such as solitary fibrous tumors (SFTs). We compared the expression levels of FOXO1, RB1, ROS markers and several signal transduction factors between SCLs and SFTs. SCLs expressed decreased levels of FOXO1 and RB1, whereas SFTs showed no change. Both tumor types exhibited increased markers of ROS; however, nuclear localization of phosphorylated p38 was significantly more frequent in SCLs than that in SFTs, suggesting p38 MAPK activation by oxidative stress. SFTs showed lower p38 MAPK activity and higher β-catenin expression, implying that oxidative stress was caused by increased cellular proliferation stress. Finally, CAFs and MFBs showed changes similar to those observed in SCLs. Overall, tumors with monoallelic 13q14 deletion showed shared molecular signatures that might be associated with pathogenesis.

## Introduction

Spindle cell/pleomorphic lipomas (SCLs), cellular angiofibromas (CAFs) and mammary-type myofibroblastomas (MFBs) are rare benign mesenchymal tumors with monoallelic 13q14 deletion [1–3]. These tumors share similar histopathological and immunohistochemical features; however, molecular events induced by this genetic alteration remain unclear. Two tumor suppressor genes, RB1 and FOXO1, are located in 13q14. While RB1 is a well-established tumor suppressor, FOXO family of transcription factors are associated with diverse functions such as cell cycle regulation, differentiation, apoptosis, DNA repair and reactive oxygen species (ROS) detoxification [4–6]. Specifically, FOXO1 was shown to be associated with alveolar rhabdomyosarcoma as a result of chromosome alteration and is considered as a tumor suppressor [4].

In a previous study, we reported a CAF case with monoallelic 13q14 deletion. The tumor expressed decreased levels of FOXO1 and RB1 as well as increased levels of oxidative stress markers, 8-hydroxy-2′-deoxyguanosine (8-OHdG) and 4-hydroxy-2-nonenal (4-HNE). Furthermore, the p38 mitogen-activated protein kinase (p38 MAPK) pathway, which is often induced by ROS, was activated in that CAF case [7].

While the molecular mechanism underlying tumorigenesis associated with monoallelic 13q14 deletion is not known, based on these findings, we hypothesized that SCLs, CAFs and MFBs would share common molecular signatures involving FOXO1 expression, ROS status and p38 MAPK activity. To examine our hypothesis, we conducted a series of comparative studies between tumors with monoallelic 13q14 deletion, SCLs, CAFs and MFBs, and those without 13q14 deletion, such as solitary fibrous tumors (SFTs), aggressive angiomyxomas (AAs), dermatofibrosarcoma protuberans (DFSP), well-differentiated liposarcoma (WLS), myxoid liposarcoma (MLS) and pleomorphic liposarcoma (PLS) which are frequently considered in differential diagnosis. We analyzed clinicopathological characteristics and performed immunohistochemistry to assess the expression levels of FOXO1, RB1 and oxidative stress markers, the p38 MAPK activity and other signal transduction pathways associated with pathogenesis.

## Methods

### Case Selection

Representative hematoxylin and eosin-stained slides of cases were reviewed for case selection. The majority of cases were derived from the consultation files of one of the authors (M. H.), which included 52 SCLs, 3 CAFs, 3 MFBs, 36 SFTs and 2 AAs. In addition, 3 SCLs, 1 CAF, 4 SFTs, 2 AAs, 11 DFSP, 12 WLS, 10 MLS and 4 PLS were retrieved from the surgical pathology files of other institutions. Several aspects of clinical, histological, immunohistochemical and cytogenetic information on a CAF sample were previously reported [7]. Since the SFT harbor inversion of chromosome 12q13 resulting in NAB2-STAT6 fusion gene, detection of STAT6 by immunohistochemistry has become a diagnostic tool for SFT. In present study, 91% (32/35) of SFT were positive for STAT6. The study protocol was approved by the Institutional Review Boards of University of the Ryukyus, Okinawa, Japan (No. 791, June 2, 2015) and University of Occupational and Environmental Health, Fukuoka, Japan (H27-029, June 23, 2015). All procedures performed in this study were carried out in accordance with the Declaration of Helsinki.

### Histopathological Analysis

The tumors were processed, stained with hematoxylin and eosin and were analyzed using immunohistochemistry. Primary antibodies used in this study were α-smooth muscle actin (Dako, Glostrup, Denmark), CD34 (Dako), desmin (Dako), Ki-67 (Dako), S-100 (Dako), RB1 (Abcam, Cambridge, United Kingdom), p16 (Abcam), STAT6 (Santa Cruz, Dallas, Texas), FOXO1 (Cell Signaling Technology, Boston, Massachusetts), phosphorylated p38 (Cell Signaling Technology), β-catenin (Millipore, Billerica, Massachusetts), 8-OHdG or 4-HNE (both from Japan Institute for the Control of Aging, Shizuoka, Japan), and the details of the staining methods were described in the previous report [7]. The appropriate positive and negative controls were included in each staining. The immunoreactivity and positive extent of the markers were semiquantitatively scored using a similar method as that reported by Sangoi et al. [8]. In brief, the immunoreactivity was graded as negative, weak, moderate, or strong. The percentage of tumor cells with each marker was semiquantitatively scored as 0 (0%–9%), 1+ (10%–49%), 2+ (50%–89%) and 3+ (≥90%). For each antibody, the immunoreactivity of more than weak and the percentage score of tumor cells more than 1+ were judged as positive. Only nuclear positive signals for RB1, FOXO1, 8-OHdG, p16, phosphorylated p38 and β-catenin were used for scoring.

### Fluorescence In Situ Hybridization Analysis

Fluorescence in situ hybridization (FISH) analysis was performed using a chromosome 13 (13q14)-specific probe (Poseidon™ repeat-free FISH probes, Kreatech Diagnostics, Amsterdam, The Netherlands), which include RB1 gene but not FOXO1 gene, according to the manufacturer’s instructions and as described previously [7].

### Statistical Analysis

Data were analyzed by two-tail Mann-Whitney U-test. A *P* value of <0.05 was considered statistically significant for all analyses.

## Results

### Clinical Findings of Tumors with Monoallelic 13q14 Deletion

The summary of clinicopathological parameters of tumors with monoallelic 13q14 are shown in Table 1. The SCLs affected 45 males and 8 females; two cases were unknown. The median age was 62 years (range, 21–87 years). The most common affected sites were the head and neck, followed by the trunk. The CAFs affected 3 males and 1 female, and the median age was 53.5 years (range, 49–69 years). The CAF from female was in the vagina, whereas the CAFs from the males were in the groin, testis and scrotum. The MFBs affected 1 male and 2 females, with a median age of 64 years (range, 54–65 years). The MFBs from the females were in the groin and knee, whereas the MFB from the male was in the mammary gland.

**Table 1:** Summary of clinicopathological features of specimens analyzed in this study

	SCL	CAF	MFB	AA	SFT	DFSP	WLS	MLS	PLS
Sex									
Male	45	3	1	1	24	7	8	5	1
Female	8	1	2	3	16	5	4	5	3
Age(y)									
< 50	11	1	0	2	13	8	2	5	0
≧50	42	3	3	2	27	4	10	5	4
Median	62.0	53.5	64.0	44.0	58.5	44	58.5	49.5	66
Range	21–87	49–69	54–65	28–57	9–84	3–68	40–79	33–81	61–92
Site									
Head and neck	24	0	0	0	11	2	0	0	1
Shoulder	10	0	0	0	2	1	1	0	0
Chest wall	1	0	0	0	2	2	0	0	0
Pleura	0	0	0	0	6	0	0	0	0
Mammary gland	0	0	1	0	0	0	0	0	0
Abdominal wall	0	0	0	0	1	1	0	0	0
Lung	0	0	0	0	2	0	1	0	0
Retroperitoneum	0	0	0	0	5	0	2	0	0
Kidney	0	0	0	0	2	0	1	0	0
Pelvic cavity	0	0	0	0	2	0	0	0	0
Back	8	0	0	0	1	3	0	0	0
Buttock	0	0	0	0	2	0	0	2	0
Extremities	11	0	1	0	1	3	7	8	3
Genital region	1	4	1	4	1	0	0	0	0
Etc	0	0	0	0	2	0	0	0	0

*SCL*, spindle cell/pleomorphic lipoma; *CAF*, cellular angiofibroma; *MFB*, myofibroblastoma; *SFT*, solitary fibrous tumor; *AA*, aggressive angiomyxoma; *DFSP*, dermatofibrosarcoma protuberans; *WLS*, well-differentiated liposarcoma; *MLS*, myxoid liposarcoma; *PLS*, pleomorphic liposarcoma

### FISH for 13q14

All SCLs were examined by FISH for 13q14. Among a total of 55 SCLs, monoallelic 13q14 deletion was confirmed in 43 cases (78%), and 12 cases unsuitable for FISH or negative for monoallelic 13q14 deletion were excluded for subsequent analyses.

### RB1 and FOXO1 Expression in Tumors with or Without Monoallelic 13q14 Deletion

**Fig. 1:**
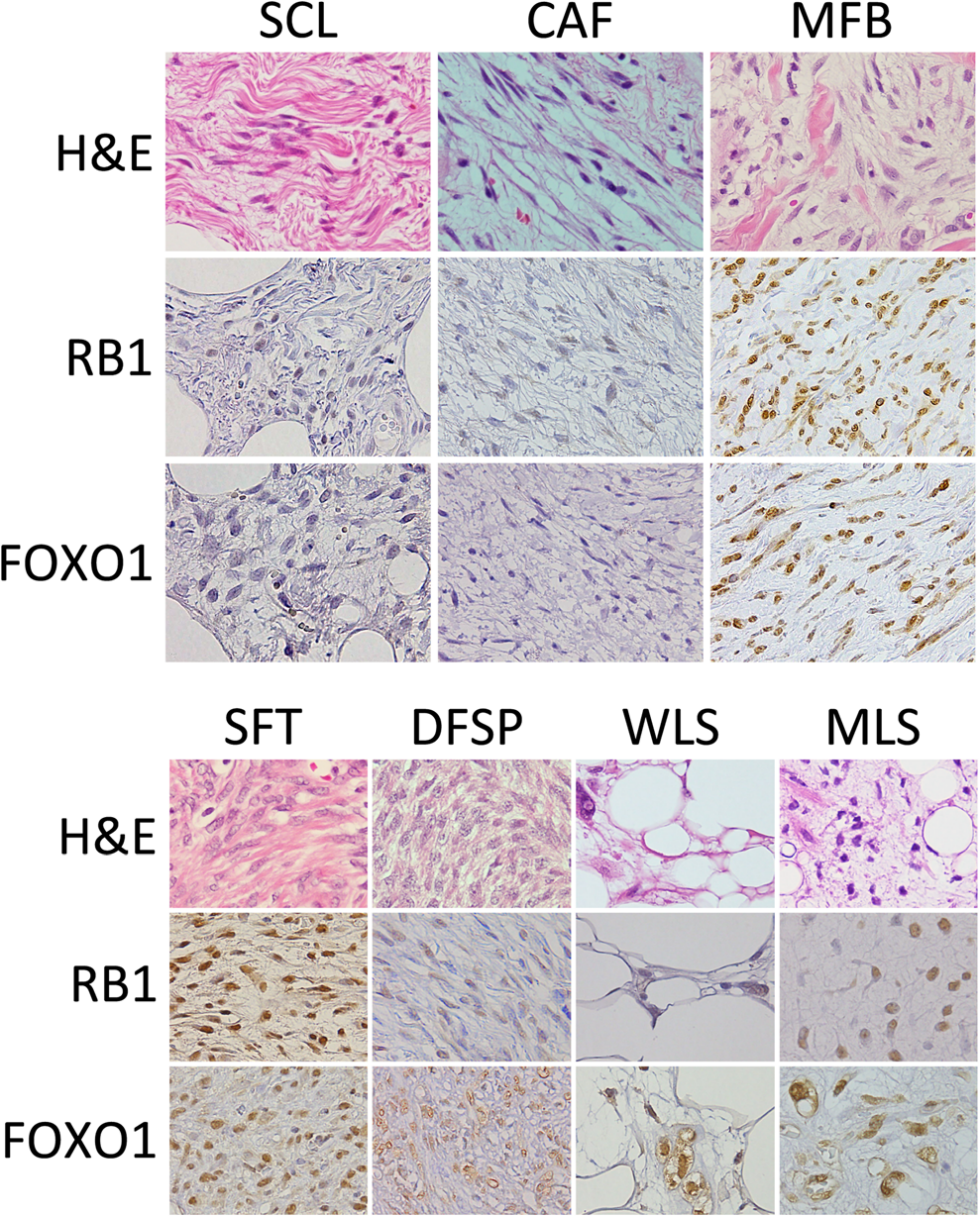
Representative images of tumor specimens stained for hematoxylin and eosin, immunohistochemically stained for RB1 and FOXO1 are shown. SCL: spindle cell/pleomorphic lipoma, CAF: cellular angiofibroma, MFB: myofibroblastoma, SFT: solitary fibrous tumor, DFSP: dermatofibrosarcoma protuberans, WLS: well-differentiated liposarcoma, MLS: myxoid liposarcoma. SCL and CAF showed decreased expression of RB1 and FOXO1, whereas other tumors exhibited various expression levels of either marker

RB1 and FOXO1 expression was evaluated because these genes are encoded in 13q14 region. Nuclear RB1 expression was confirmed in 29 of 42 (69%) SCLs; however, the majority exhibited either weak or moderate immunoreactivity. RB1 expression was also detected in 1 of 4 CAFs and all MFBs (3/3). SFTs showed higher rates of positive RB1 expression (32/40; 80%) than those observed in SCLs, and the immunoreactivity to RB1 in SFTs tended to be higher than that observed in SCLs. Finally, PLSs (0/4) showed no expression and WLSs (3/11) demonstrated low frequency, whereas the others including AAs (3/4), DFSPs (5/11) and MLSs (8/10) were positive for RB1 (Table 2, Fig. 1).

**Table 2:** Summary of immunohistochemical results of tumor specimens analyzed in this study

	SCL	CAF	MFB	SFT	AA	DFSP	MLS	WDLS	PLS
**RB1**									
• Positive rate	29/42	1/4	3/3	32/40	3/4	5/11	8/10	3/11	0/4
• Positive extent									
3+	1	0	1	21	1	0	0	0	0
2+	6	0	1	8	2	0	0	0	0
1+	22	1	1	3	0	5	8	3	0
0	13	3	0	8	1	6	2	8	0
• Immunoreactivity									
Strong	2	0	0	8	0	0	1	0	0
Moderate	14	2	2	18	2	1	3	4	0
Weak	19	1	1	8	2	5	5	4	0
None	7	1	0	6	0	5	1	3	4
**FOXO1**									
• Positive rate	16/30	1/4	3/3	31/36	2/4	5/11	9/10	9/11	0/4
• Positive extent									
3+	1	0	0	10	1	0	7	3	0
2+	4	0	1	16	1	1	1	3	0
1+	11	1	2	5	0	4	1	3	0
0	14	3	0	5	2	6	1	2	4
• Immunoreactivity									
Strong	1	0	0	10	0	0	7	3	1
Moderate	4	1	1	14	2	5	3	2	0
Weak	12	1	2	7	1	1	0	5	1
None	13	2	0	5	1	5	0	1	2
**8-OHdG**									
• Positive rate	36/42	4/4	2/3	31/40	2/4	6/7	7/10	5/11	4/4
• Positive extent									
3+	24	4	1	15	1	1	1	0	1
2+	6	0	1	10	1	0	2	4	0
1+	6	0	0	6	0	5	4	1	3
0	6	0	1	9	2	1	3	6	0
• Immunoreactivity									
Strong	7	1	1	0	0	5	4	8	3
Moderate	18	3	0	21	2	1	1	1	0
Weak	12	0	2	12	1	0	3	2	0
None	5	0	0	7	1	1	2	0	1
**4-HNE**									
• Positive rate	34/42	4/4	2/3	34/40	3/4	3/4	8/10	10/12	4/4
• Positive extent									
3+	27	2	1	27	0	2	0	2	1
2+	4	2	1	1	2	0	1	4	0
1+	3	0	0	6	1	1	7	4	3
0	8	0	1	6	1	1	2	2	0
• Immunoreactivity									
Strong	6	2	1	7	0	1	5	9	2
Moderate	14	1	0	20	1	1	3	2	1
Weak	14	1	2	7	2	1	0	1	1
None	8	0	0	6	0	1	2	0	0
**phosphorylated p38**									
• Positive rate	28/42	4/4	2/3	15/40	3/4	3/10	6/10	2/12	0/4
• Positive extent									
3+	14	4	1	0	0	0	0	1	0
2+	10	0	1	3	2	0	2	0	0
1+	4	0	0	12	1	3	4	1	0
0	14	0	1	25	1	7	4	10	4
• Immunoreactivity									
Strong	2	1	1	0	0	4	5	3	3
Moderate	19	2	1	2	3	1	2	6	0
Weak	7	1	0	14	1	0	2	3	0
None	14	0	1	24	0	5	1	0	1
**p16**									
• Positive rate	42/42	4/4	3/3	29/38	3/4	6/6	3/10	3/6	
• Positive extent									
3+	22	3	2	8	1	3	0	0	ND
2+	15	1	1	12	2	2	1	0	ND
1+	5	0	0	9	0	1	2	3	ND
0	0	0	0	9	1	0	7	3	ND
• Immunoreactivity									
Strong	23	2	2	7	0	6	0	0	ND
Moderate	15	1	0	12	3	0	3	0	ND
Weak	4	1	1	12	1	0	1	3	ND
None	0	0	0	7	0	0	6	3	ND
***β*** **-catenin**									
• Positive rate	15/42	0/4	1/3	28/40	3/4				
• Positive extent									
3+	0	0	1	18	1	ND	ND	ND	ND
2+	4	0	0	7	2	ND	ND	ND	ND
1+	11	0	0	3	0	ND	ND	ND	ND
0	27	4	2	12	1	ND	ND	ND	ND
• Immunoreactivity									
Strong	0	0	0	11	0	ND	ND	ND	ND
Moderate	9	1	1	12	2	ND	ND	ND	ND
Weak	10	0	0	5	1	ND	ND	ND	ND
None	23	3	2	12	1	ND	ND	ND	ND

*SCL*, spindle cell/pleomorphic lipoma; *CAF*, cellular angiofibroma; *MFB*, myofibroblastoma; *SFT*, solitary fibrous tumor; *AA*, aggressive angiomyxoma; *DFSP*, dermatofibrosarcoma protuberans; *WLS*, well-differentiated liposarcoma; *MLS*, myxoid liposarcoma; *PLS*, pleomorphic liposarcoma. SCL and SFT were highlighted by bold text for comparison

Nuclear FOXO1 expression was detected in 16 of 30 (53%) SCLs. The majority of FOXO1-positive cases exhibited weak immunoreactivity. FOXO1 was expressed in 1 of 4 CAFs, and in all the MMF specimens analyzed; however, none of those showed a strong FOXO1 immunoreactivity. The majority of SFTs (31/36: 86%) were positive for FOXO1, and FOXO1 immunoreactivity tended to be higher than that observed in SCLs. Although PLSs (0/4) showed no expression, AAs (2/4), DFSPs (5/11), WLSs (9/11) and MLSs (9/10) expressed FOXO1 (Table 2, Fig. 1).

### Oxidative Stress Markers in Tumors with or Without Monoallelic 13q14 Deletion

**Fig. 2:**
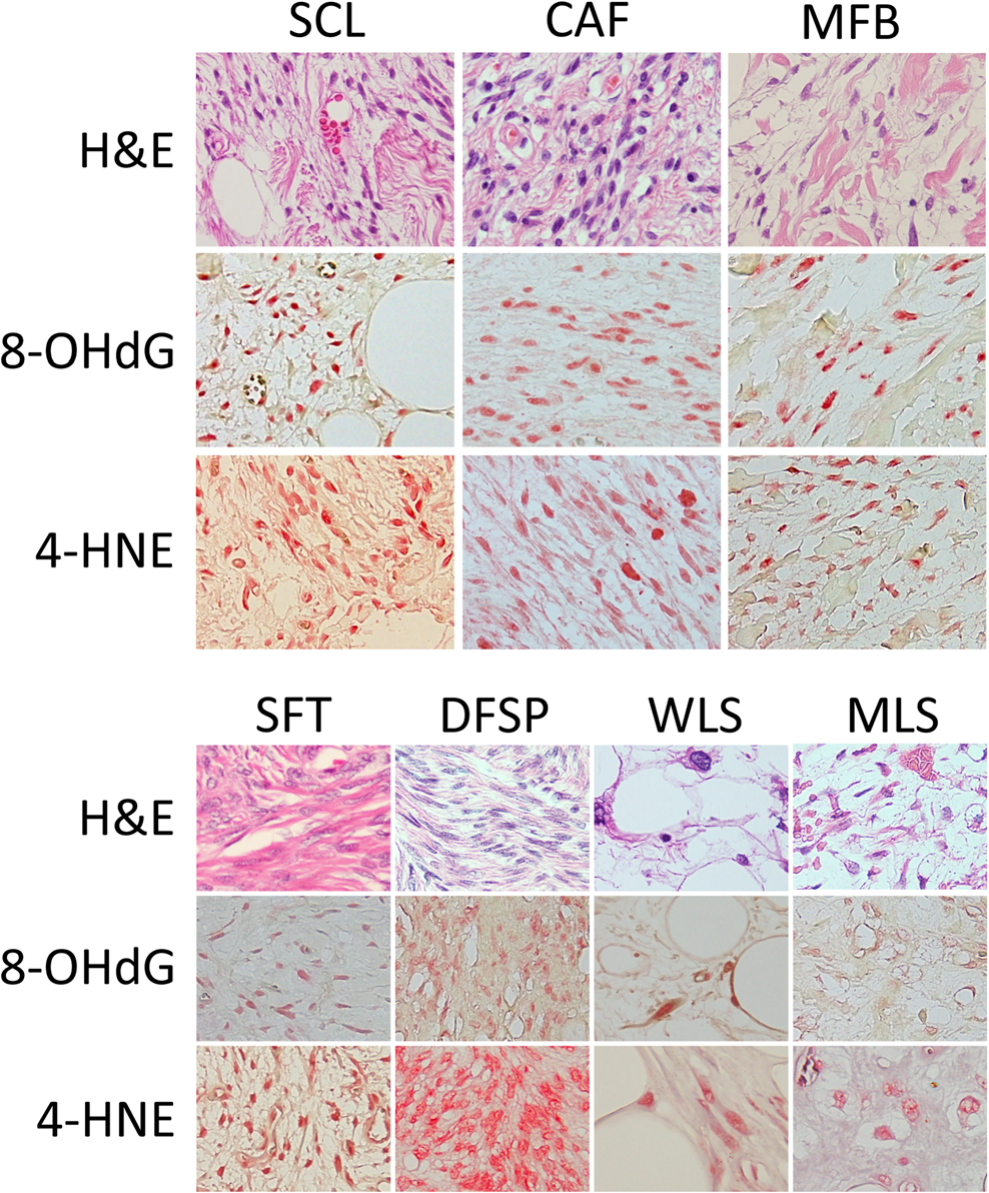
Representative images of tumor specimens stained for hematoxylin and eosin, immunohistochemically stained for 8-hydroxy-2′-deoxyguanosine (8-OHdG) and 4-hydroxy-2-nonenal (4-HNE) are shown. SCL: spindle cell/pleomorphic lipoma, CAF: cellular angiofibroma, MFB: myofibroblastoma, SFT: solitary fibrous tumor, DFSP: dermatofibrosarcoma protuberans, WLS: well-differentiated liposarcoma, MLS: myxoid liposarcoma. Specimens from tumors with monoallelic deletion of 13q14 as well as those without 13q14 deletion demonstrated increased expression of markers of oxidative stress

Two oxidative stress markers were evaluated in this study. The majority of SCLs were positive for both 8-OHdG (36/42: 86%) and 4-HNE (34/42: 81%). The majority of CAFs and MFBs also exhibited positive staining for these two markers. Similarly, SFTs also showed increased expression of 8-OHdG (31/40: 78%) and 4-HNE (34/40: 85%). Finally, most of the AAs, DFSPs, WLSs, MLSs and PLSs indicated high positivity for both markers (Table 2, Fig. 2).

### p38 MAPK and β-Catenin Signaling in Tumors with or Without Monoallelic 13q14 Deletion

**Fig. 3:**
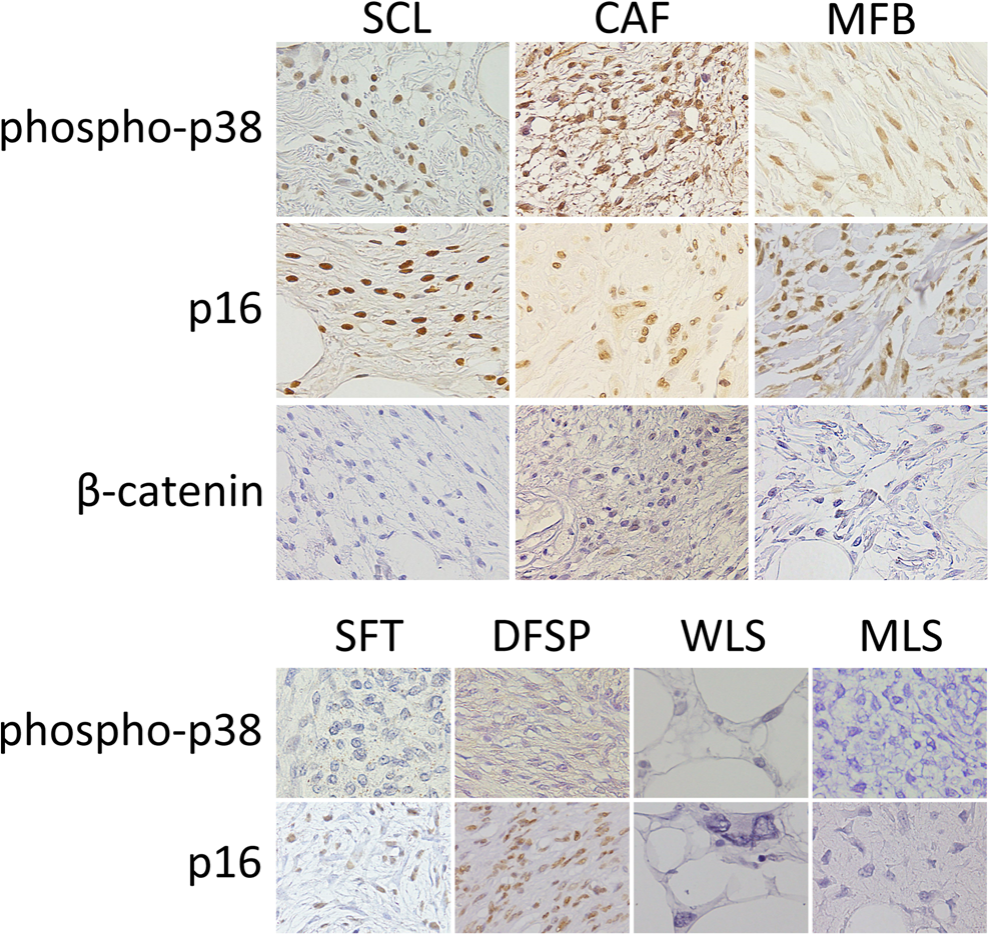
Representative images of tumor specimens immunohistochemically stained for phosphorylated p38, p16 and β-catenin are shown. SCL: spindle cell/pleomorphic lipoma, CAF: cellular angiofibroma, MFB: myofibroblastoma, SFT: solitary fibrous tumor, DFSP: dermatofibrosarcoma protuberans, WLS: well-differentiated liposarcoma, MLS: myxoid liposarcoma. SCL, CAF and MFB specimens were positive for phosphorylated p38 and p16 in the nucleus and showed decreased expression of β-catenin. In contrast, SFT specimens exhibited decreased phosphorylated p38 and p16 expression levels. The others showed various expression levels of these markers

Nuclear phosphorylated p38 localization was demonstrated in 28 of 42 (67%) SCLs. The majority of those positive for phosphorylated p38 showed moderate immunoreactivity. A positive nuclear phosphorylated p38 was also seen in all 4 CAFs, as well as 2 out of 3 MMFs. Of a total 40 SFTs, 15 cases showed nuclear phosphorylated p38 localization (15/40; 38%); however, the extent and immunoreactivity of phosphorylated p38 in SFTs tended to be lower than those observed in SCLs. Finally, most of AAs and MLSs were positive for nuclear phosphorylated p38, whereas DFSPs, WLSs and PLSs showed low positive rate (Table 2, Fig. 3).

Because p16 is one of the important signal transduction factors downstream from p38 MAPK pathway, we compared expression of p16 between SCLs and SFTs. All SCLs expressed p16 (42/42: 100%), and among them, 38 cases were either moderately or strongly immunoreactive for p16. Of SFTs, 76% (29/38) were positive for p16, whereas p16 immunoreactivity varied across specimens (Table 2, Fig. 3).

Nuclear β-catenin localization was observed in 15 of 42 (36%) SCLs; the β-catenin immunoreactivity was either moderate or weak in these cases. No nuclear β-catenin was detected in CAFs, whereas 1 out of 3 MMFs expressed nuclear β-catenin. In contrast, the majority of SFTs (28/40: 70%) were positive for β-catenin which tended to be expressed stronger than that observed in SCLs (Table 2, Fig. 3).

## Discussion

SCLs, CAFs and MFBs share a common genetic defect: monoallelic 13q14 deletion [1–3]. While two tumor suppressor genes, RB1 and FOXO1, are encoded in this region, the precise molecular pathogenesis of these tumors is unknown. To date, there are relatively few reports investigating the pathogenesis of these tumors. Chen et al. reported that soft tissue tumors with monoallelic 13q deletions, such as SCLs and CAFs as well as the majority of MMFs did not express RB1, which might lead to subsequent tumor development. Although the mechanism underlying the decreased level of RB1 expression is yet unclear, they suggested a mutation on the remaining allele or an epigenetic change that affects the expression of RB1 [9]. Magro et al. reported deletion of FOXO1 in mammary- and vaginal-type MFBs by FISH analysis and proposed that tumors with 13q14 deletion belong to a single entity with a continuous spectrum of different morphological presentations [10]. However, FOXO1 expression level in these tumors has not been reported. In the present study, we compared the expression of RB1 and FOXO1 between SCLs (tumor with monoallelic 13q14 deletion) and SFTs (that without 13q14 deletion), because the number of cases is sufficient for statistical analysis between these tumors. Contrary to our prediction, the rate of RB1 positivity was not different between SCLs and SFTs, however, the extent of positive signal and immunoreactivity to RB1 in SCLs were lesser than those in SFTs. Because we used 13q14 FISH probe which included RB1 region, one signal in the present FISH experiment suggested loss of one RB1 gene. Therefore, the extent and immunoreactivity of RB1 in SCLs may reflect the gene dosage of RB1, i.e., one allele of RB1 gene by monoallelic deletion. The difference of RB1 positivity between present study and the study by Chen et al. (2012) could have been due to the different anti-RB1 antibody used. The positive rate as well as both positive extent and immunoreactivity to FOXO1 in SCLs were lower than those in SFTs (53% versus 86%, *p* < 0.05). Because the FISH probe used in the present study did not include FOXO1 region, and FOXO1 gene is approximately 7,8 Mb apart from RB1 gene, the monoallelic 13q14 deletion did not necessarily demonstrate the loss of one allele of FOXO1 gene. The expression control of FOXO1 may have a similar expression mechanism of FOXP2, one of FOX gene family, and its mutation is associated with a neurodevelopmental defect in acquisition of spoken language. FOXP2 undergoes random monoallelic expression (RMAE), which results in three distinct expression statuses: expression of “both alleles” in some cells and expression of either the maternal or paternal allele in other cells. FOXP2 expression of “both alleles” is controlled under cis-regulatory element that map to 3 Mb away from the FOXP2 gene locus. Therefore, a deletion of the cis-regulatory element, which is demonstrated in a patient with developmental verbal dyspraxia, results in FOXP2 haploinsufficiency (monoallelic expression) [11]. Because cis-regulatory element of FOXO1 in 13q14 is unknown and whether FOXO1 operates RMAE is yet unclear, further study regarding the mechanism of FOXO1 expression is required to understand the impact of monoallelic 13q14 deletion. The present study suggested that monoallelic 13q14 deletion in SCLs affects the expression of both RB1 and FOXO1. The number of CAFs and MFBs were limited in our study; however, both CAFs and MFBs showed low expression levels of RB1 and FOXO1. Based on the downregulation of both these tumor suppressors, we hypothesize that these events may be contributing to a shared pathogenic mechanism of tumors with monoallelic 13q14 deletions, as proposed by Magro et al. [10].

In a previous study, we reported a CAF patient with monoallelic 13q14 deletion and loss of FOXO1 expression, which was accompanied by increased expression of oxidative stress markers [7]. We also showed the activation of p38 MAPK pathway, which is often induced by oxidative stress [12, 13]. The present study confirmed the increased oxidative stress and subsequent p38 MAPK activation in SCLs. These findings suggested that the loss of FOXO1 function in tumors with monoallelic 13q14 deletion induces oxidative stress, leading to the activation of p38 MAPK signaling. The role of p38 MAPK signaling in soft tissue tumors remains controversial. Two groups reported that therapy using SRC family kinase inhibitors, or artesunate, antimalarial drug, induced growth inhibition of rhabdomyosarcoma through the activation of p38 MAPK [14, 15], whereas another group reported that p38 MAPK might play a role in induction of human telomerase reverse transcriptase in sarcomas, suggesting an association between p38 MAPK and malignant transformation [16].

In this study, we found higher p38 MAPK activity in SCLs than that in SFTs (67% versus 38%, *p* < 0.05). While the rate and intensity of oxidative stress marker expression were similar between SCLs and SFTs, the responses to oxidative stress might differ between them. Given the low rate of FOXO1 positivity in SCLs, the response to oxidative stress might be impaired, resulting in increased ROS levels that lead to p38 MAPK activation. Since p16 is one of the important signal transduction factors downstream from p38 MAPK pathway, the high rate of p16 positivity in SCLs also suggested p38 MAPK activation. CAFs and MFBs showed similar results to those in SCLs. These findings suggested that tumors with monoallelic 13q14 deletion express decreased FOXO1 levels, leading to increased ROS accumulation that result in the activation of p38 MAPK pathway.

Despite similar levels of ROS between SCLs and SFTs, FOXO1 positivity rate was higher in SFTs than SCLs, suggesting that the response to ROS elicited by FOXO1 in SFTs is intact. In addition, p38 MAPK activity was lower in SFTs than SCLs; again, these results might reflect an intact FOXO1 mediated antioxidant response in SFTs. In general, oxidative stress in cancer cells is aggravated by oncogene expression, increased metabolic activity, hypoxic conditions, chronic inflammation and damaged mitochondrial function [17–19]. As neither oxidative stress nor p38 MAPK activity in SFTs has been reported to date, a literature search did not result in any information on the antioxidant response in SFT; however, the present study suggests that increased oxidative stress in SFTs induces normal FOXO1 activation.

Hoogeboom et al. reported that binding of FOXO family members to β-catenin inhibits β-catenin/T cell factor (TCF) transcriptional activity, ultimately resulting in suppression of the Wnt signaling [20], implicating FOXO as tumor suppressor. Therefore, we analyzed nuclear β-catenin translocation and hypothesized that tumor with monoallelic 13q14 deletion would show increased nuclear β-catenin translocation. However, in contrast to our hypothesis, β-catenin expression and its nuclear localization were lower in SCLs than SFTs (36% versus 70%). These results implied that normal functions of adenomatous polyposis coli (APC) were preserved in SCLs, which lead to proper proteasomal degradation of β-catenin. Based on these results, we proposed that the Wnt/β-catenin pathway is not related to the pathogenesis in tumors with monoallelic 13q14 deletion.

In the present study, the number of CAF and MFB cases was few, therefore, the comparison of positivity of each marker between these tumors and SCLs showed limited correlation. Furthermore, the present immunohistochemical analysis demonstrated that p38 MAPK activation was induced by increased ROS, but the resulting biological effects such as cell proliferation, apoptosis and cell senescence remain unclear. Further molecular analyses using fresh specimen is needed.

In summary, the tumors with monoallelic 13q14 deletion show decreased expression level of FOXO1, which is encoded in the 13q14 region, and increased levels of oxidative stress markers as well as higher p38 MAPK activity. The above results lead us to hypothesize that those tumors with monoallelic 13q14 deletion share molecular signatures involving the increase in the oxidative stress markers that are induced by decreased FOXO1 expression; furthermore, increased oxidative stress result in the activation of p38 MAPK pathway. The present study suggests these molecular events are associated with tumorigenesis caused by 13q14 deletion.

## Abbreviations

SCL: Spindle cell/pleomorphic lipoma
CAF: Cellular angiofibroma
MFB: Mammary-type myofibroblastoma
SFT: Solitary fibrous tumor
AA: Aggressive angiomyxoma
DFSP: Dermatofibrosarcoma protuberans
WLS: Well-differentiated liposarcoma
MLS: Myxoid liposarcoma
PLS: Pleomorphic liposarcoma
